# Fanning the Flames of Inflammaging: Impact of Monocyte Metabolic Reprogramming

**DOI:** 10.20900/immunometab20200025

**Published:** 2020-07-01

**Authors:** Brandt D. Pence

**Affiliations:** 1 School of Health Studies, University of Memphis, Memphis, TN 38152, USA; 2 Center for Nutraceutical and Dietary Supplement Research, University of Memphis, Memphis, TN 38152, USA

**Keywords:** aging, monocyte, mitochondria, glycolysis, inflammation, immunometabolism, inflammaging

## Abstract

Monocytes are circulating innate immune cells that are functionally dysregulated during aging. However, while metabolic regulation of innate immune cell function is now well-established, only a handful of studies have examined this in the context of aging. In a recent article published in *Aging Cell*, Saare et al. observe comprehensive metabolic reprogramming of otherwise unstimulated monocytes isolated from older adults. These cells display increased glucose uptake and dysregulation of mitochondrial function, concomitant with activation of signaling pathways contributing to increased inflammation. These findings suggest a mechanism whereby metabolic reprogramming in aged monocytes contributes to chronic low-grade inflammation and open new avenues of investigation into the biological underpinning of inflammaging.

Monocytes are circulating cells of the innate immune system which participate in a breadth of host defense and inflammatory processes, including antigen presentation, phagocytosis, inflammatory cytokine and chemokine production, and extravasation into tissue followed by differentiation to macrophages or dendritic cells [1]. As a principal circulating inflammatory cell, monocytes have long been speculated to be major contributors to the age-associated chronic inflammatory state often termed “inflammaging” [2]. Because inflammaging is thought to underlie the bulk of age-related chronic diseases, monocytes are potential therapeutic targets for strategies meant to ameliorate aging-related disease.

Within the context of aging, multiple previous studies have found profound monocyte and macrophage dysfunction, including increased basal inflammation, impaired inflammatory activation, altered phagocytosis, and impaired migration/chemotaxis [3]. In recent years, a variety of cellular metabolic programs have been shown to be linked to immune cell functions, and this has been comprehensively reviewed elsewhere [4]. However, immunometabolic studies have not been extensively undertaken in the aging field, and whether aging triggers shifts in immune cell metabolic programs is not well-understood.

In 2018, my laboratory was the first to demonstrate that aging impaired mitochondrial function in monocytes when we showed that isolated human classical monocytes displayed reduced mitochondrial respiratory capacity using Seahorse assays [5]. Now, a new paper by Saare et al. in *Aging Cell* [6] replicates and extends our initial finding, substantially expanding our knowledge of aging and monocyte immunometabolism using a variety of strategies for interrogating metabolic reprogramming.

Using whole genome RNA sequencing, Saare et al. demonstrated a gene transcription pattern in isolated CD14^+^ classical monocytes from older individuals which was suggestive of a decrease in mitochondrial function and oxidative phosphorylation and, concomitantly, an increase in glycolytic energy production. Subsequent experiments found increased reactive oxygen species (ROS) production and enhanced glucose uptake in unstimulated monocytes from older adults. Interestingly, aged monocytes displayed somewhat abrogated inflammatory responses as measured by lipopolysaccharide (LPS)-induced phosphorylation of STAT3, and these cells also failed to upregulate glucose uptake in response to LPS stimulation to the same extent as monocytes from younger individuals.

In addition to increased ROS and decreased mitochondrial spare capacity, the authors of this paper noted trends toward increased mitochondrial mass and reduced mitochondrial membrane potential in monocytes from older adults, and using these assays in tandem demonstrated that mitochondrial membrane potential was substantially decreased on a per mitochondrion basis. Overall, classical monocytes from older adults appeared to have a degree of mitochondrial dysfunction which may increase reliance on glycolytic metabolism during a quiescent state, and this may cause the increase in basal inflammatory activity in monocytes demonstrated here and in previous studies [7].

Interestingly, the results in Saare et al. are not wholly consistent with previous monocyte immunometabolism work from my laboratory. While we previously found impaired mitochondrial respiratory capacity in classical monocytes isolated from older adults (as in the current paper), we also found no differences in mitochondrial membrane potential, mitochondrial mass, or ROS production in unstimulated monocytes [5]. Further, in a previous paper, we demonstrated no differences in glycolytic activation in unstimulated or LPS-stimulated classical monocytes from older adults [8], which is in contrast to the present study. However, in those studies we did note impaired inflammatory activation in aged monocytes, consistent with data on LPS-stimulated STAT3 signaling in Saare et al.

Several experimental design differences may have caused these divergent results. The subjects in Saare et al. were substantially older (average age 79–82 years across experiments) compared to subjects in our previous studies (average age 65–67 years across experiments) [5,8]. Our studies also had a highly racially diverse sample and somewhat smaller sample sizes than the present paper, which has the potential to increase variability and obscure small differences in cellular metabolism. Additionally, glucose metabolism was measured by flow cytometric quantification of the glucose analog 2-NBDG in Saare et al., while we utilized Seahorse assays to measure glycolysis via quantification of extracellular acidification [8], which may contribute to disparate findings.

Inflammatory activation during LPS stimulation was decreased in both the present study and our previous work [8], which is suggestive of a relationship between mitochondrial dysfunction and impaired inflammatory activation in these cells. Supporting this, a recent study published in *Brain, Behavior and Immunity—Health* demonstrated decreased LPS-induced expression of tumor necrosis factor-α with pharmaceutical inhibition of mitochondrial Complexes I-IV [9]. However, Complex IV inhibition also increased interleukin-6 production, suggesting the links between mitochondrial dysfunction may be specific to certain cytokines. Additionally, these assays were performed in whole blood, and therefore cell-specific contributions to these responses are in need of additional study.

The results from the research presented here also lead to questions as to other mechanisms whereby mitochondrial dysfunction might contribute to age-related monocyte functional impairments. For example, monocytes have been shown to switch to mitochondrial oxidative phosphorylation for energy production during inflammatory activation when glucose is not present [10]. While we recently found no evidence that aged monocytes have impairments in this type of metabolic flexibility [11], this may yet prove to be impaired in monocytes from substantially older subjects such as those used in the present study. Additionally, contributions of mitochondrial dysfunction to age-related impairments in phagocytosis, chemotaxis, and other monocyte immune functional responses is still an open question.

The present findings by Saare et al. suggest a potential mechanism whereby mitochondrial dysfunction in aged monocytes leads to higher glucose utilization and, consequently, increased inflammatory cytokine production in an otherwise unstimulated state. Within the context of aging, the contributions of cellular senescence to an overall pro-inflammatory state are now well known. Much of this results from the production by senescent cells of the senescence-associated secretory proteome (SASP), which includes a variety of pro-inflammatory cytokines, chemokines, and growth factors [12]. Of note, recent evidence suggests that non-classical monocytes exhibit hallmarks of senescence, including both mitochondrial dysfunction and increased SASP production [13], and non-classical monocyte proportions in the circulation are increased during aging [5,7]. While mitochondrial dysfunction in both Saare et al. and our previous study was examined only in classical monocytes, it is possible to speculate that these metabolic impairments result from increasing cellular senescence in these monocytes, which may dispose them to transition to the non-classical phenotype. This is deserving of additional study.

In summary, Saare et al. [6] present convincing data that aging leads to a metabolic program in monocytes which is consistent with an increased pro-inflammatory state. This work opens a variety of avenues for follow-up experiments interrogating the biological causes for this metabolic reprogramming, and the delineation of other functional impacts of monocyte mitochondrial dysfunction is also an intriguing area of future investigation. Open questions currently include the mechanisms underlying mitochondrial dysfunction in monocytes, as well as the contributions of metabolic reprogramming to age-related functional impairments in other monocyte responses such as phagocytosis, antigen presentation, and anti-pathogen immunity. Additionally, the impact of metabolic reprogramming and mitochondrial dysfunction on monocyte metabolic flexibility, monocyte phenotype transitions, and macrophage and dendritic cell differentiation are worthy of additional exploration. The present paper suggests a metabolic program in aged monocytes (Figure 1) which leads to inflammaging and, ultimately, to age-related chronic disease. Reprogramming of monocyte metabolism is therefore a promising therapeutic strategy for treating age-associated diseases.

## Figures and Tables

**Figure 1.**
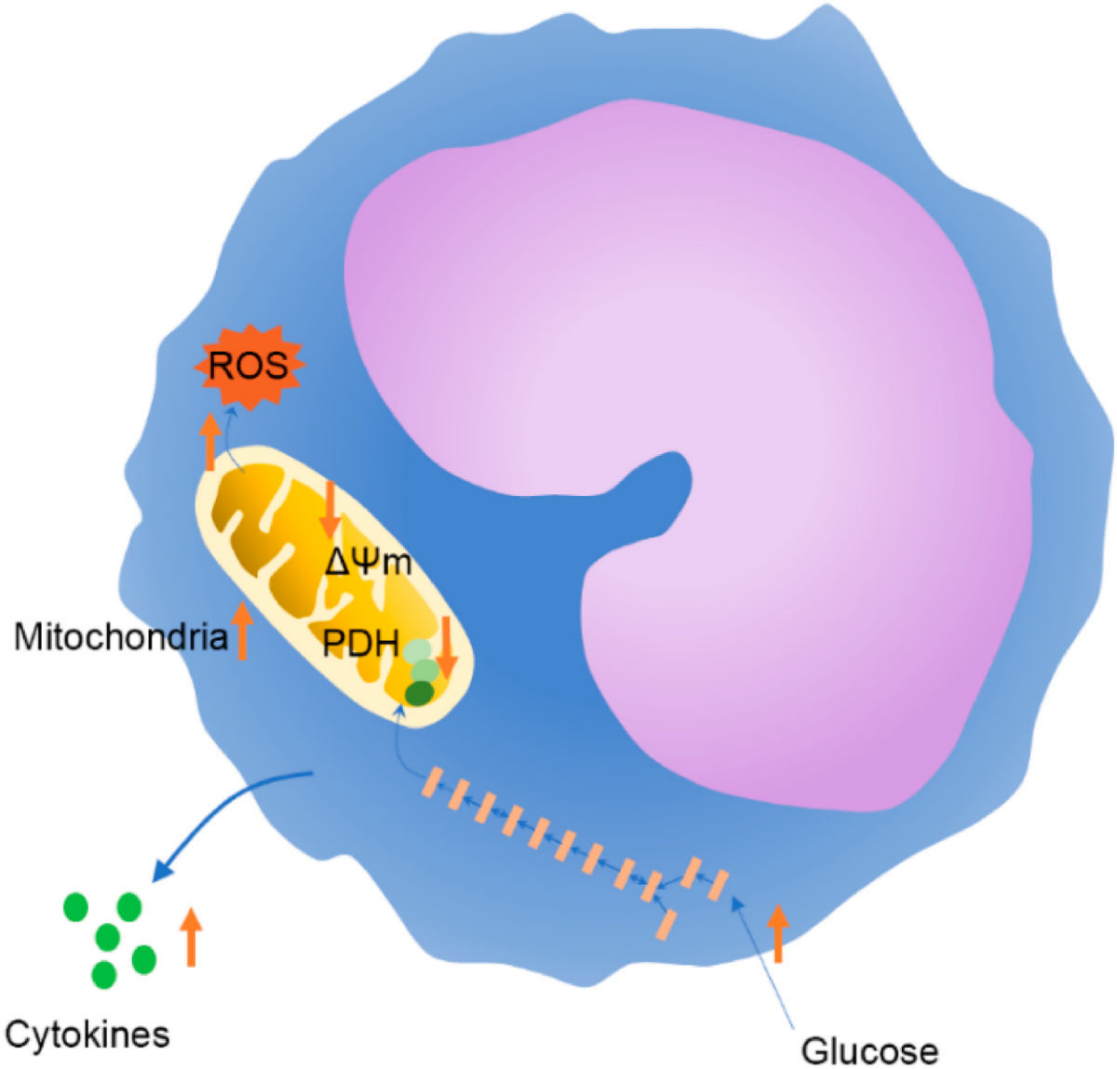
Proposed changes in monocyte metabolism during aging based on findings from Saare et al. [6]. Aging increased glucose uptake, mitochondrial mass, and reactive oxygen species (ROS) production. Aged monocytes also displayed gene expression changes consistent with decreased pyruvate dehydrogenase (PDH) activity and had reduced mitochondrial membrane potential (ΔΨm). Increased phosphorylation of STAT3 and activated mTOR signaling were also suggestive of the potential for increased inflammatory cytokine release in unstimulated monocytes from older adults.

## ABBREVIATIONS

LPS: lipopolysaccharide
ROS: reactive oxygen species
